# Adaptation “from below” to changes in species distribution, habitat and climate in agro-ecosystems in the Terai Plains of Nepal

**DOI:** 10.1007/s13280-019-01202-0

**Published:** 2019-06-10

**Authors:** Jessica P. R. Thorn

**Affiliations:** 1 Department of Environment and Geography, University of York, Room 313, 290 Wentworth Way, Heslington, York YO10 5NG UK; 2 African Women in Climate Change Science Fellow, African Climate and Development Initiative (ACDI), University of Cape Town, Geological Sciences Building, Upper Campus, Level 6, 13 Library Road, Rondebosch, Cape Town, 7700 South Africa; 3 Department of Ecosystem Science and Sustainability, Colorado State University, Room A126A, Campus Delivery 1476, Fort Collins, CO 80523 USA; 4 Department of Zoology, University of Oxford, 1a Mansfield Rd, Oxford, OX1 3SZ UK

**Keywords:** Autonomous adaptation, Biodiversity, Climate change, Ecosystem services, Land-use change, Local ecological knowledge

## Abstract

Recent land-use and climatic shifts are expected to alter species distributions, the provisioning of ecosystem services, and livelihoods of biodiversity-dependent societies living in multifunctional landscapes. However, to date, few studies have integrated social and ecological evidence to understand how humans perceive change, and adapt agro-ecological practices at the landscape scale. Mixed method fieldwork compared observed changes in plant species distribution across a climatic gradient to farmers’ perceptions in biodiversity and climate change in rice-cultivated farms. In contrast to the global context, farmers in the Terai Plains of Nepal are acutely aware of high levels of change observed in the last 10 years, and incrementally adapt as new invasive species emerge (93%), the incidence and severity of pest/diseases increase (66%), genetic diversity of indigenous varieties erodes (65%), forest habitats diminish (98%), irrigation water declines (60%), and wildlife ranges shift. Twenty-five changes in climate were reported by 97.5% of farmers to reduce provisioning services and food self-sufficiency, and increase exposure to waterborne pathogens, heat stress, and human or livestock mortality. The study illustrates the need for financial and institutional supports at all levels to strengthen agro-ecological practices, upscale Information Communication Technology for extension services, clarify tenure agreements, and safeguard natural ecosystems to slow biodiversity loss. Existing incentives to conserve, restore, or sustainably manage ecosystems offer lessons for other societies undergoing rapid change.

**Electronic supplementary material:**

The online version of this article (10.1007/s13280-019-01202-0) contains supplementary material, which is available to authorized users.

## Introduction

Recent shifts in the distribution and composition of species are occurring in parallel with changes in temperature, precipitation, and ecosystem services provisioning across landscapes (IPCC 2018). Concurrently, humans play a major role in re-engineering social–ecological systems in desirable ways, affecting species composition and diversity, and the intensity and frequency of weather-related hazards (Leadley et al. 2010). Yet, few studies empirically investigate how the management of indigenous communities living in multifunctional landscapes is changing in response to biodiversity and climate change (Salick and Ross 2009). Many rural farming populations are particularly unique, in that they have stewarded and directly depended on some of the Earth’s most unique biodiversity for thousands of years (Guneratne 2002). Their vulnerability differs to other systems where services are more likely to be substitutable, and they often adapt in ways that are unaided by external agencies, nor necessarily reflected in formal policies. Local knowledge and practices remain the foundation for any response, and are often the only interventions to reduce risks (Boissiëre et al. 2013). The existing literature has primarily focused on adaptation strategies that can be implemented on a large-scale in developed countries (Howard 2009). What is needed is a better understanding of impacts of compounding risks, localized adaptive responses, and factors influencing farmers’ choices to sustainably manage agrobiodiverse landscapes. In this context, recent global (Nakashima et al. 2012), regional (UNFCCC 2010), and national (Salick and Ross 2009) calls have been made for detailed interdisciplinary case studies to illuminate human adaptations in response to biodiversity and climate change, particularly in Least Developing Countries.

In recent years, there has been a rapid expansion of the literature seeking to define, measure, and value ecosystem services (e.g., MEA 2005; IPBES 2016; Haines-Young and Potschin 2017). These efforts are underpinned by the rationale that quantifying ecosystem services can lead to better planning and inform management strategies. Here, ecosystem services are defined as the various benefits people accrue from ecosystems, which contribute directly to human well-being and economic wealth (Constanza et al. 1997). We employ provisioning (e.g., food, fuelwood), regulating (e.g., water), supporting (e.g., biodiversity), and cultural services (e.g., aesthetic value)—as categorized by the first large-scale and widely recognized ecosystem service assessment (MEA 2005).

An increasing number of studies address the drivers and effects of agricultural land-use changes on ecosystem services (Denu et al. 2016), and the consequences of climate change for agricultural livelihoods (Lal et al. 2016). Other studies that investigate climate impacts on species diversity and abundance suggest that in the upcoming decades, climate change could surpass habitat destruction as the greatest global threat to biodiversity (Leadley et al. 2010; Chen et al. 2011). For example, large portions of Amazonian rainforest could be replaced by tropical savannahs (Lapola et al. 2009). However, climate change ecology is still an emerging field. Potential impacts are typically assessed using bioclimatic envelope or dynamic vegetation models, while few assessments are at the landscape level (Bellard et al. 2012). To date, research has seldom integrated social and ecological data to establish how humans adapt agro-ecological practices in response to biodiversity and climate changes (Howard 2009).

Using the case of highly biodiversity-dependent farming communities across four landscapes in the Terai Plains of Nepal, this paper is guided by the following questions: What are observed changes in plant species distribution across a climatic gradient? What are farmers’ perceptions of species distribution and habitat change? What are farmers’ perceptions of climate-driven changes, and do they differ across regions? What adaptations to land management are autonomously adopted at the landscape level in response to biodiversity and climate change? Study findings could inform the allocation of resources through the Climate Investment Fund, National and Local Adaptation Plans of Action, as well as conservation and livelihood programs.

Nepal is a recent example of a country experiencing changes in biodiversity, cultural knowledge, and climate change. Operating mainly as an agrarian economy, the majority of the workforce (73.9%) depends on subsistence agriculture (Government of Nepal 2016). The country also has a high conservation value: being home to eight of the world’s ten highest mountains (UN General Assembly 2015), holding 2.3% of the total world freshwater supply, and stewarding an extensive forest cover of 39.6% (5.83 m ha). Significantly, the flora and fauna of the region constitute a biodiversity hotspot that requires research attention and protection (Bhattacharjee et al. 2017).

However, the country has recently experienced shifts in biodiversity, cultural knowledge, and climate change, particularly in the Terai Plains (hereon the Terai). Following the passing of the 1964 Land Act, where productive land was made freely available to people from the Mid-hills, major biodiversity losses occurred. Dichlorodiphenyltrichloroethane (DDT) was introduced in 1965, resulting in a steep decline in malaria incidence (Dhimal et al. 2014). To boost rice production (1.7–3.5 m between 1961 and 2015), and cater for a doubling population (35–62% between 1952 and 2011), 0.1 m ha of forests were cleared between 1950 and 1986, while newly sunken boreholes depleted shallow aquifers (USAID 2009; Government of Nepal 2011). Simultaneously, the cultural–demographic profile of the population substantially shifted from small pockets of Tharu (the original indigenous tribal population, and the largest ethnic minority in Nepal comprising over 2000 subdivisions), to a mixture with Brahmin, Chettri, Indian migrants, and other castes (Guneratne 2002).

Today, the Terai is often referred to as the “food basket” or “granary” of the country, given its fertile soils from flat alluvial deposits. Despite its relatively small area, the Terai accounts for 68% of Nepal’s agricultural output, produces 30.7% of the national GDP from agricultural production, and constitutes 43% of cultivated land, 21% of land cover, and 70% of industries (Government of Nepal 2016). Notwithstanding its richness, communities are highly sensitive and vulnerable to global environmental change (Government of Nepal 2009). This is in part due to the country’s undulating topography, high levels of poverty (25.2% live on US $0.50 day^−1^), as well as technological and institutional constraints to effective response mechanisms (World Bank 2015; International Labour Organization 2017). While a few scholars have studied the traditional knowledge systems of populations in the Terai, many regions remain understudied (Guneratne 2002). Studies have typically analyzed meteorological data (Malla 2008), or focused on particular strategies to climate change, such as flood and drought management, precision agriculture, crop and livelihood diversification, or early warning systems (Bhatta and Aggarwal 2016a; Devkota et al. 2014; Ghimire et al. 2010). Consequently, there remains a dearth of scientific research on the subject of human adaptation to biodiversity change.

## Materials and methods

### The study area

**Fig. 1:**
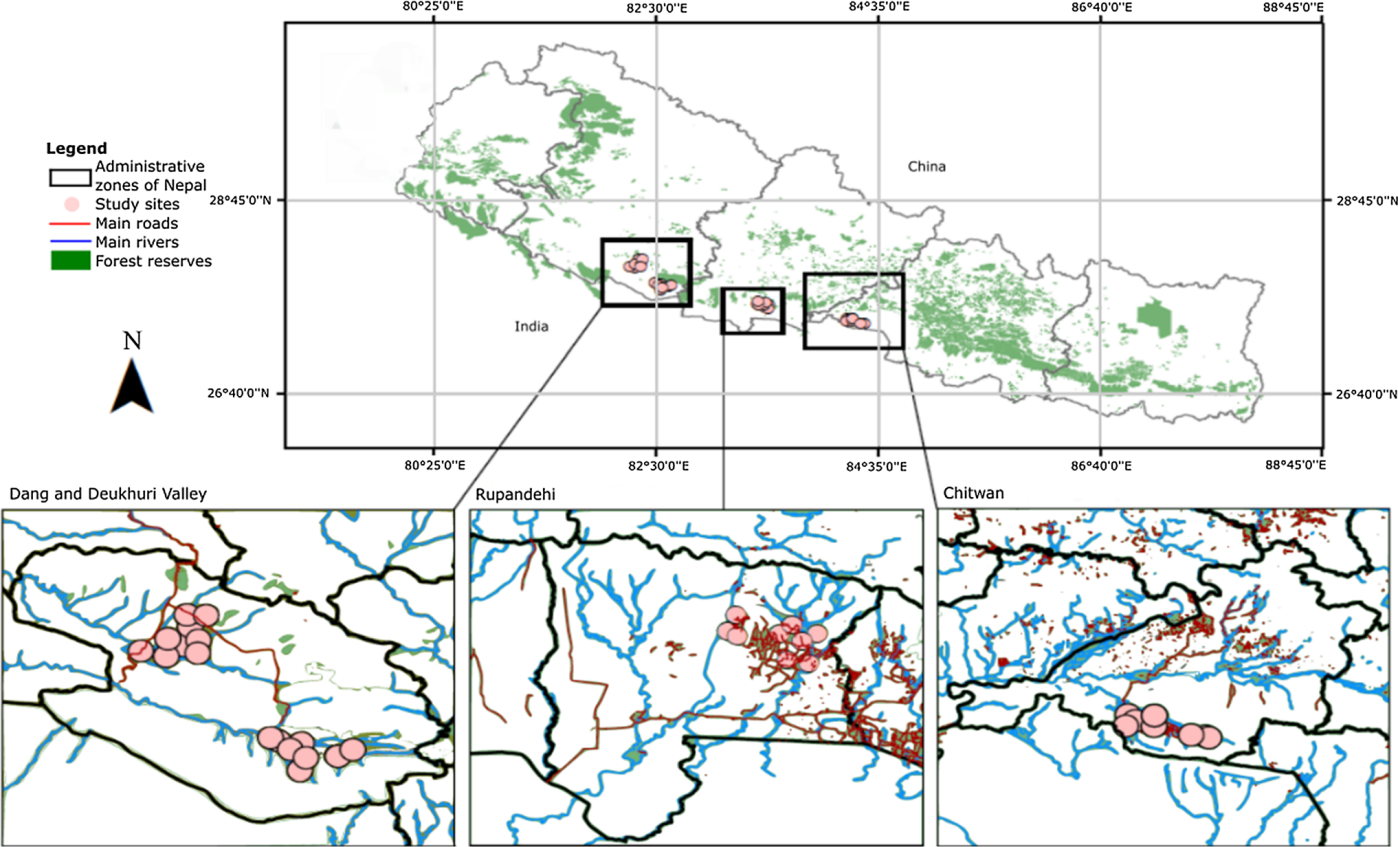
Map of study area in the Central and Western zones of the Terai Plains of Nepal (*n *= 40 villages, *n *= 427 households). The Terai is the lowland region in the Southern Nepal. Sampling was carried out in 22 village district committees (VDCs) and 40 wards: (1) four VDCs in Madi Valley, Chitwan district (N27°28.305’ E084°17.244′, 204masl), (2) six VDCs in Rupandehi district (N27°35.414′ E083°31.180′, 138masl), (3) six VDCs surrounding Gohari, Dang district (N27°50.783′ E082°30.068′, 256masl) (referred to hereafter as Dang), and (4) six VDCs in the Deukhuri Valley, Dang district (N28°03.086′ E082°18.712′, 597masl) (Deukhuri)

Study sites spanned four climatically distinct landscapes in the Central and Western zones of the Terai—with the Himalayan Churia foothills to the North, and India to the South (Fig. 1). Situated in the warm-temperate Indo-Malayan Tropical Monsoon zone, the mean annual temperature is 24.6 °C (min = 18.2 °C, max = 31 °C), while rainfall ranges from 1000 to 2100 mm year^−1^. The Terai is one of the country’s five physio-geographic zones, stretching 1360 km. It differs from the higher regions in the rest of the country due to its unique climate (i.e., tropical and subtropical, compared to temperate in the hills and snowy alpine), and agricultural commodities produced (i.e., predominantly cereals, fruits and vegetables) (Chalise et al. 1996). Rice crops were studied as they are a major staple commodity driving rural employment, and constitute a significant proportion of consumers’ protein and caloric intake. Rice is grown predominantly for local consumption (49–79 kg person^−1^ year^−1^), and has historically been a food habit of Nepalese people (World Bank 2015).

### Field sampling

Data was collected at household and landscape levels between May and September 2012 and 2014, during the summer monsoon season when 80% of the annual precipitation falls (Bhattacharjee et al. 2017). To identify how climate-driven changes alter plant species diversity and abundance, space-for-time substitution was used. This is a widely recognized method in the field of ecology to infer past or future trajectories from contemporary spatial patterns (Pickett 1989). Regions were compared that represent a precipitation gradient from East (wetter) to West (drier), which may reflect possible trajectories of the latest IPCC assessment (2014) (Appendix S1, S2). That is, model ensembles suggest that by this mid century, the Indo-Gangetic Plains might experience more variable rainfall, fewer growing days, and drier, hotter conditions. Such changes could lead to ~ 50 % reduction in rice yields (IPCC 2014). Precipitation was the main variable considered, given its importance for crop production, and that local communities’ easily perceive changes in irrigation needs (Niles and Mueller 2016). A fundamental assumption of the space-for-time substitution approach is a lack of correspondence between other climatic and nonclimatic features, and their relative importance. Consequently, biophysical and economic data was also considered.

In each climatic region ten plots were identified using regional 2012 topographic maps of Nepal (1:25 000) from the Ministry of Land Management, Cooperatives and Poverty Alleviation, and East View Cartographic Inc. (USA). Additionally, local partners were consulted. Sites were distributed within a 200 km^2^ block across the hydro-shed catchment (i.e., Rewu, Tinau, Rapti, and Bogai rivers). The selection criteria for the vegetation sampling included farms that were rice-cultivated, and had no chemical fertilizer applied in the previous month. Furthermore, the land manager had to have lived in the area for at least 10 years (since 2002), been actively cultivating the land for at least 1 year, and was locally recognized as having a deep knowledge of the vegetation. Local authorities’ or elders’ consent and input into the study design was required prior to commencing the study.

### Ecological surveys

Standardized vegetation sampling procedures were used to collect unmanaged plant specimens (Bridson and Forman 1998). Forty farms (10 m × 10m square grids) were surveyed over 2 months (July and August) 06h00–10h00 (one sample species^−1^ farm^−1^). Plots were located on land up to 35°, using a north–south, east–west orientation. The stem, leaf, fruit and seed of all vascular aboveground plant specimens found in the plot were collected, processed, identified, and photographed, with the help of taxonomic experts. Specimens were then stored in the National Herbarium and Plant Laboratories in Kathmandu, for future reference. To identify Scientific and English names of species, the nomenclature of Press et al. (2000) was followed, and verified using previous studies. Qualitative ecological inventory interviews were carried out where the vegetation sampling took place. The manager responsible for the farm at the time of the survey was asked about the names and uses of the chosen species (*n *= 75).

### Household surveys and key informant interviews

Next, we ran a semi-structured questionnaire consisting of 152 questions for circa 90 min, covering the following components: socioeconomic information; farming system; household characteristics; biodiversity and climate change; food security and health; water regulation and supply; pest and disease regulation; and adaptations to management (Appendix S3). It was pretested with 40 respondents considered representative of the population. Approximately 100 interviewees were selected in each of the four landscapes (*n *= 426), stratified by age (25–67 years), sex (72.5% male, 27.5% female), caste (*n *= 9), and livelihood (*n *= 14), although stratification was restricted by the site selection criteria. Survey results were then discussed for triangulation. All interviews were conducted in Nepali, except when this was not the respondents’ first language, in which case local farmers assisted in interpretation, and plain language was used. A lead surveyor supervised and quality checked three trained enumerators to ensure precision and consistency in sampling, data collection, and data entry. Finally, to infer policy implications, key informants representing a range of sectors, institution types and scales of operation were interviewed (*n *= 174, Appendix S1).

### Data analysis

Plant taxonomic absolute and proportional abundances were calculated, as was diversity using the Shannon–Wiener diversity index. Here, *s* is the number of individuals, and *p*_*i*_ is the relative proportion of individuals belonging to total (*i*) individuals (Shannon and Weaver 1949).

$$ {\text{Shannon}} {-} {\text{Wiener}}:H^{\prime} = - \sum\limits_{i = 1}^{s} {\left( {p_{i} * \, \ln \, p_{i} } \right)} $$

To compare how community composition differed across all and within each climatic region, the study used a one-way analysis of variance and Pearson’s Chi-squared goodness-of-fit tests, after count data were logtransformed, using the car (Fox et al. 2018) and lattice (Sarkar 2017) packages. Trend stability analysis over 20 years (1991–2011) was run for mean monthly maximum and minimum temperature, and total annual rainfall, using 720 meteorological datasets. Data was sourced from three regional synoptic stations (Government of Nepal 2012) averaging 19 km from the study sites, and the literature (Paudel et al. 2014, Appendix S4, S5). Interview data were analyzed using descriptive statistics and narrative situational analysis. This approach is an extension of grounded theory, where in an effort to understand complex social-ecological systems, the situation becomes the fundamental unit of analysis (Clarke et al. 2018). Audio recordings were transcribed, and the content of narratives were qualitatively interpreted (Krippendorf 2004). Data were analyzed in R Studio V.3.1.1 (R Development Core Team 2017).

## Results

### Farming systems

Across the Central and Western Terai farming systems are highly susceptible to biodiversity and climate-driven changes that affect their agricultural systems (Table 1). The mean landholding size of farms is 5.12 ± 4.78 ha household^−1^, ranging from smallholder (min. 0.72 ha) to commercial sized plots (max. 19.46 ha). Twenty-three crop types are cultivated for an average of 6.56 ± 1.63 years. Rice (*Oryza sativa* L.) is the main crop grown in 88% of fields in the summer monsoon season, producing on average 3.63 ± 1.7t ha^−1^ season^−1^. This figure is slightly higher than the national average of 2.74t ha^−1^ and the regional average of 3.08t ha^−1^ in South Asia (2014). In some areas (45% of fields), rice is relay cropped with lentils (*Lens culinaris* Medic). Maize (*Zea mays* L.) is the second most widely cultivated cereal crop (65%), followed by wheat (*Triticum aestivum* L.) (48%). Mustard (*Brassica juncea*) is the main oilseed crop, cultivated in 38% of cases. The selection of crops depends on various factors including crop water requirement (e.g., soya bean), phosphorus fixation (e.g., banana), nitrogen fixation (e.g., lentil), altitude or temperature range (e.g., cabbage, cauliflower, some varieties of radish), or whether it is a high-value crop (e.g., aloe vera, peppermint). The average household owns 19.64 Total Livestock Units (as defined by FAO 2011)—most commonly goat (53%) and buffalo (50%), followed by cows (28%), poultry (28%), and oxen (20%). Vast tracts of land in the Terai remain unirrigated, leaving 74% of the study population depending predominantly on rain-fed irrigation—similar to the national average of 72% (World Bank 2017). Small-scale stand-alone irrigation water sources play an important part of rural life [e.g., hand-drawn tube-wells (26%), electric tube-wells (3%)], while rainwater tanks and ponds are rare. In the rainy season, most irrigation water (67%) is allocated from small or medium surface rivers, using canals (30%), electric pumps (15%), or rivers (27%). Drinking water is typically extracted using hand-pumped shallow tube wells (90%), sunk to 30.7 ± 19.7 feet. Access to irrigation water is generally communal (86%), compared to drinking water, which is generally private (78%).

**Table 1:** Site characteristics across climatic regions

Parameter	Site characteristics	Chitwan, Madi Valley (wettest)	Rupandehi	Deukhuri Valley, Dang	Dang, near Ghorahi (driest)
Climate	Total annual rainfall (mean over 20 years) (mm)	2666	1623	1575	1598
	Mean annual temp (mean over 20 years) (°C)	23.75	24.82	22.59	21.84
Population	Male respondents (%)	70	90	80	50
	Female respondents (%)	30	10	20	50
	Age of respondents (year)	44.1 ± 7.89	54.2 ± 10.29	41.3 ± 7.51	40.6 ± 8.29
	Age household head (%) ≤ 40 year	40	0	30	60
	Age household head (%) 41–64 year	60	70	70	40
	Age household head (%) ≥ 65 year	0	30	0	0
	Size of community (hh)	164.56 ± 28.48	190 ± 31.39	140.67 ± 43.75	149.2 ± 23.81
	Time lived in the community (year)	6.83 ± 0.65	39 ± 5.05	35.67 ± 6.89	6.4 ± 0.54
	Tharu (%)	20	20	20	70
	Gurung (%)	0	20	0	0
	Brahmin (%)	30	40	30	10
	Chettri (%)	10	10	30	0
	Dalit (%)	20	0	10	0
	Other (%)	20	10	10	20
	Relocated for marriage (%)	16.67	NA	NA	40
	Migrated from hilly regions (%)	16.67	NA	NA	10
	Procured land (%)	16.67	NA	NA	0
	Born in community (%)	50	NA	NA	5
Yield	Yield—rice (ton ha^−1^)	3.21 ± 0.57	4.12 ± 0.65	5.16 ± 0.79	3.2 ± 0.31
	Crop yield—household (%)	76.75 ± 5.51	69.88 ± 11.09	86.25 ± 8.53	51.44 ± 6.17
	Crop yield—sale (%)	5.51 ± 5.51	30.13 ± 11.09	13.75 ± 8.53	33.19 ± 5.76
	Crop yield—fodder/other (%)	0	0	0	2.88 ± 2.32
	Total Livestock Units	13.82	11.6	31.23	21.9
Livelihoods	Reliable income 9–12 months/year	70	30	NA	NA
	Food self-sufficient months	10.2	12	NA	NA
Land management	Area cultivated of all crops (ha)	3.6 ± 0.82	5.78 ± 2.33	5.9 ± 1.56	5.21 ± 1.35
	Owned land (%)	70 ± 0.13	87.7 ± 0.1	64.7 ± 0.08	81.7 ± 28.42
	Land ownership inheritance: procurement: government	90:10:00	80:10:10	70:20:10	90:10:00
	Fallowing (% of population)	50	75	0	13
	Crop rotation (% of population)	71	100	NA	90
	Improved varieties in last 10 year (% of population)	100	80	30	60
	Terracing (% of population)	70	40	50	20
	Pesticide use (% of population)	90	80	90	80
Water management	Area irrigated (ha)	1.11 ± 0.39	2.37 ± 1.18	1.74 ± 0.82	0.95 ± 0.31
	Cultivated land that is irrigated(%)	29	24	36	29
	Shallow tube well depth (feet)	23 ± 3.47	46.17 ± 11.07	42 ± 10.54	7.32 ± 0.17
	Communal irrigation (%)	80	85.71	87.5	88.89
	Private irrigation (%)	20	14.29	12.5	11.11
	Ground water irrigation (%)	40	62.5	22.22	11.11
	Surface water irrigation (%)	60	37.5	77.78	88.89
	Borehole irrigation (% of total)	60	22	0	20
	Electric pump irrigation (% of total)	40	0	11	10
	Canal irrigation (% of total)	0	67	11	40
	Direct flow from river (% of total)	0	11	78	20
	Electric borehole (% of total)	0	0	0	10

Values represent the mean ± SE (*n *= 40 villages, *n *= 426 respondents). The significance of caste is that it may determine one’s education, income, occupation, and social standing, thereby influencing knowledge systems related to biodiversity management and agricultural practices. Fallowing was defined as cultivated land that is not seeded for one or more growing season, and crop rotation was defined as the alternation of subsistence, cash and green manure/cover crops with different characteristics, cultivated on the same field during successive years. Livelihoods refers to respondents’ perceptions of reliable income derived from both agricultural and nonagricultural sources. Information was collected in land management and socioeconomic surveys (Appendix S3). The year 2002 was the reference year for 10 years’ prior (*y* year, *t* tonne)

### Household characteristics

Villages typically contain 159.84 ± 15.37 households, who have lived there for 23.6 ± 3.88 years. Fifty-three per cent of the study population were born in the villages where they currently reside, 29% relocated to their spouses’ residence when marrying, while 18% migrated from the hilly regions. Farmers produce largely for household subsistence purposes (65%) using family labor, while 27% of produce is sold, or used for fodder and gifted (8%). Financial capital to buffer farmers from shocks is limited: 50% stated they have reliable income derived from agriculture and other sources 9–12 months year^−1^, 17% 6–9 months year^−1^, and 27% 3–5 months year^−1^. Farmers are generally unaware of the market value of produce, and lack means to transport goods in bulk—relying on bicycles (83%) or motorbikes (50%). A large number of respondents in the Terai are relatively well-educated, compared to other regions in Nepal, but these high education levels have contributed to a growing remittance economy (Bhatta and Aggarwal 2016b). Formal education levels peak generally at secondary school level, with all accessing a primary school within < 5 km distance. All households have access to mobile phones, 90% to radio, 80% to television, and 50% to internet. Forty per cent of farmers rent under various rental agreements (*adhiya*), while only 13% have procured land. Across the year, most farmers depend on agriculture, and related activities, for their livelihood and income. However, 32% have 13 additional livelihood activities including: teaching (15%), foreign employment (8%) (e.g., labor, hospitality, tractor-rental, and shop or hotel ownership), and 5% work in construction, bee-keeping, wagon driving, river mining, aquaculture, technical extension work, or own a medical center. An increasing number of farmers (23%) are involved in member-controlled community-level enterprises (e.g., women’s groups, farmer’s cooperatives).

### Observed changes in species distribution across a climatic gradient

**Fig. 2:**
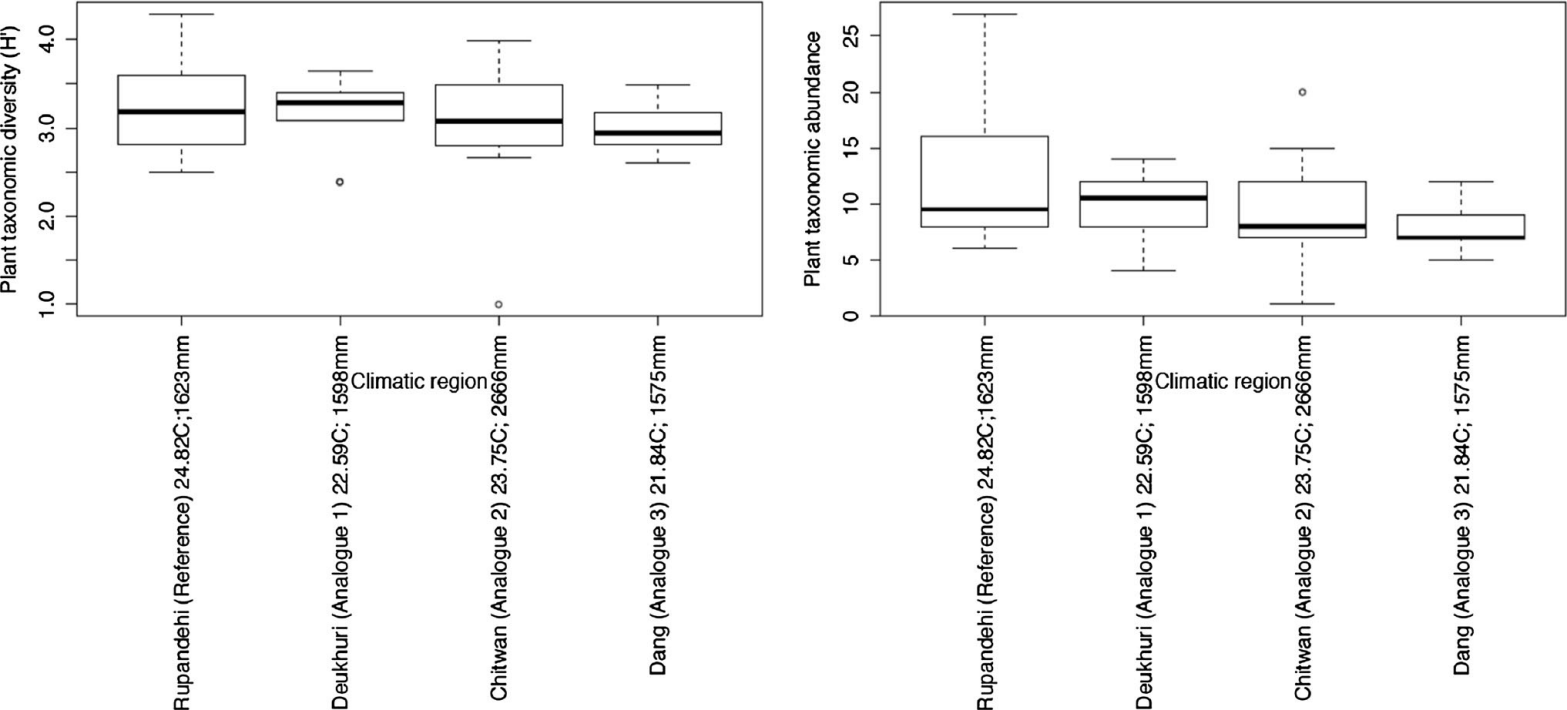
Boxplots of unmanaged plant species diversity (H’) and abundance across climatic regions. Boxplots showing the highest diversity and abundance across climatic conditions were found in Deukhuri, with a mean total annual rainfall of 1598 mm and a mean annual temperature of 22.59 °C. Values show mean (line) and standard error (bar) (*n *= 40)

Overall, 390 vascular plant specimens were collected and identified as belonging to 75 distinct plant species from 49 phylogenetic families (for a detailed species list see Appendix S6). Across all sites, species diversity (*H*′) was 3.09 ± 0.09. Significant differences in plant diversity were seen across all climatic regions (*F*_(3,36)_ = 4.5, *p *= 0.008), as well as between Rupandehi (3.22 ± 0.17) and Dang (2.99 ± 0.08) (*F*_(1,8)_ = 7.14, *p *= 0.028)—with a mean total precipitation of 48 mm and mean annual temperature 2.98 °C lower in Dang (20 year ave.). Comparatively, no significant difference was detected across climatic regions in absolute (*F*_(3,36)_ = 0.96, *p *= 0.4), nor proportional ($x_{(45)}^{2} = 44.93,p = 0.4$) plant abundance. The highest absolute abundance was found in Rupandehi (*n *= 121), followed by Deukhuri (*n *= 96), Chitwan (*n *= 94), and Dang (*n *= 79). Both plant abundance (12.1 ± 2.06 individuals site^−1^) and diversity (3.22 ± 0.17 individuals site^−1^) was the highest in Rupandehi, which displays the warmest, but not the wettest conditions. Therefore, all other things being equal, results indicate variation in precipitation could affect plant species diversity in agricultural landscapes (Fig. 2, Table 2).

**Table 2:** Comparison of unmanaged plant species diversity (*H*′) and abundance across climatic regions

Parameter	Chitwan (wettest)	Rupandehi	Deukhuri	Dang (driest)	All farms
Annual precipitation (mm)	2666	1623	1598	1575	NA
Annual temperature (°C)	23.75	24.82	22.59	21.84	NA
Plant taxonomic diversity	3 ± 0.26	3.22 ± 0.17	3.14 ± 0.14	2.99 ± 0.08	3.09 ± 0.08
Plant taxonomic abundance	9.4 ± 1.66	12.1 ± 2.06	9.6 ± 1.06	7.9 ± 0.69	9.75 ± 0.74

### Farmers’ perceptions of species distribution and habitat change

#### Invasive species

Most farmers (93%) highlighted a proliferation of invasive species and weeds in the last 10 years, particularly in hotter, more humid conditions. Weed multiplication is also attributed to the prevalence of monoculture, land clearance (weeds are often the first plants to reclaim bare land), and the unrestricted use of farmyard manure containing undecomposed seeds. Thirty-four types of weeds are found in direct-planted and transplanted rice fields; most commonly (59%) dog’s tooth grass (*Cynodon dactylon*), lantana (*Lantana camara*), sticky snakeroot (*Ageratina adenophora*), and night-flowering jasmine (*Nyctanthes arbor*-*trisis*). Weeds compete for nutrients, water, sunlight, and species dispersal.

#### Water regulation and supply

In hotter conditions, 40% fewer farmers have access to irrigation water, and 20% fewer farmers have access to drinking water. Across the study area, 60% of farmers report a decline in irrigation water available from both shallow aquifers (65%) and surface water (55%). Changes are more pronounced in the dry season when overextraction from boreholes takes place, and hard soils do not infiltrate the water. Alternatively, water availability declines when water storage infrastructure, built with low-grade materials or inadequately maintained, cannot withstand heavy rainfall. Moreover, in hotter, wetter conditions, farmers reported that quality declines by 7.1%. Water contamination also arises from excess fertilizer inputs, seasonal turbidity, and unregulated riverbed mining for building material.

#### Pests and plant hosts for fungal pathogens

Across the study area, some 66% of farmers report increases in incidences and severity of pests and diseases found on farms in the last 10 years, while 29% report a decrease. Twenty-three types of pests have the most severe impacts on yield, income, and household consumption. The most commonly cited local names of insect pests that affect 75% of rice crops include *gabaro* (cotton bollworm, *Helicoverpa armigera), kumre kira* (different species of beetles, *Coleoptera scarabaeidae*), *kalo*/*raato kagekhapate* (cockchafers, *Melolantha melolantha,* blister beetle, *Epicauta hirtipes)*, and *aarukohariyolaii* (green peach aphid, *Myzus persicae)*. More pest infestations are attributed to growing pesticide resistance, the use of chemical fertilizer and hybrid seeds, and less fallowing. Higher temperatures and humidity also affects plant hosts for fungal pathogens, likely to be late potato blight, foliar blight, rust, and rice blast.

#### Genetic diversity and erosion

In spite of many introduced crops, 65% report indigenous varieties of cultivated plants are diminishing or threatened, thereby jeopardizing agrobiodiversity of the Terai. Local cultivars are being replaced by hybrid varieties that tolerate saturated soil (e.g., *Makawanpur*-*1*), are early-maturing (e.g., *Hardinath*-*1*), late-maturing (e.g., *Makawanpur*-*1*), hardy to allow for longer storage (e.g., *TPS 2*), resistant to emerging pathogens and diseases (e.g., *Rampur Masuli*), or have high yield (e.g., *Sabitri*) or market value (e.g., *Gorakhnath*). In addition to changing preferences, it was found that in hotter conditions, 20% of farmers use more hybrid seeds. Another 30% report that habitat fragmentation also effects the persistence of indigenous varieties. For example, the areas which animals can pollinate plant species is more restricted in fragmented landscapes.

#### Wildlife populations

Farmers observe both increase and decline in wildlife populations, and a proximity effect. Those living within 5 km from protected area boundaries, or who collect fodder and fuelwood, observe greater numbers of tigers (*Panthera tigris*), elephants (*Elephas maximus*), rhinoceros (*Rhinoceros unicornis)*, blue cows (*Boselaphus tragocamelus)*, and spotted deers (*Axis axis*). The presence of these animals increases risk of crop raids, property damage, disease contraction, injury, or even death. Conversely, other farmers report wildlife populations are declining. For example, some bird species populations’ reproductive cycles are affected by toxic exposure to DDT. Fewer reptiles, and fish are found in degraded wetlands, while herbicides adversely affects amphibian, snake, and snail populations.

#### Forest habitat

As is the case in many agricultural landscapes, almost all respondents (98%) consider timber overextraction to be a major driver of biodiversity change, resulting in species’ habitat loss. Demand for fuel wood is ever-growing, with 90% of households depending on firewood for both cooking and lighting. Wood is usually collected from community forests (58%) by foot or oxcart. Other sources of energy are biogas (62.5%), dung (53%), liquefied petroleum gas (20%), or crop residue (8%). Meanwhile, only 8% have grid-connected electricity, solar power, or use batteries or kerosene lamps. Some 88% use wood for building material, which is mostly extracted from community forests (75%), near homesteads and farms (73%), or near rivers (40%). However, with increasing income levels, 68% of houses are built with a combination of local materials and procured synthetic products. Changes in forest cover, in turn, affect areas used for hunting, collecting water, nontimber forest products, and medicinal plants, and forage quality and availability.

### Farmers’ perceptions of climate-driven changes

Ninety seven per cent of study respondents observed 25 key climate changes in the last 10 years (Table 3): most frequently this included the delay of monsoon rain by 1 month (previously commencing in May/June and currently in June/July); higher temperatures in both summer and winter; and more erratic, variable rainfall in shorter periods, followed by extended dry periods. Even small changes to rainfall patterns can have major consequences throughout the growing cycle for farmers—as described by Laksmi in Amelia, Chitwan:

“Humanity is in the age of evil, when twelve suns shine. Everything is difficult. We have to live with the fear every day that our family will be swept away by the river” (12/08/2012).

Furthermore, 63.3% report that droughts are becoming more frequent, particularly during the winter months. Meanwhile, 67% experience flooding during the monsoon season—as described by Janak of Khairah, Deukhuri:

“In the past, we had slow, gradual and continuous rain for long time. Now there is a huge amount of rain at once, and then no more” (30/08/2012).

Climate unpredictability affects planning, and during heavy rainfall riverbanks breach and erode, increasing river channel depth, water velocity downstream, and diverting watercourses. In severe cases (29%), arable land is washed away, becomes unproductive, or is abandoned.

**Table 3:** Comparison of perceptions of provisioning services across climatic regions

Perceptions of biodiversity change	Chitwan (wettest)	Rupandehi	Deukhuri Valley, Dang	Dang, near Ghorahi (driest)
Main energy source—fuelwood	90	70	100	100
Main energy source—grid electricity, solar, battery or lamp	10	20	0	0
Main energy source—liquefied petroleum gas	10	40	30	0
Main energy source—human or animal biogas	60	50	60	80
Main energy source—crop residue	20	10	0	0
Main energy source—livestock feces	60	40	30	80
Building material extracted from or near river	30	40	20	70
Building material extracted from or near forest	80	70	80	70
Building material extracted from or near farm	40	60	90	100
Firewood extracted from planted forest around homestead	20	30	70	20
Firewood extracted from forest	60	60	30	80
Firewood bought in market	20	40	30	0
Houses built—with wood	90	80	90	90
Houses built—with mud	60	60	80	80
Houses built—with bricks	70	50	10	70
Houses built—with iron	40	50	60	40
Houses built—with cement	40	60	20	60
Houses built—with rocks or stone	30	30	10	70
Houses built—with reeds or bamboo	50	40	60	40

Results represent the percentage of the study population

### Cultivated and livestock provisioning services

In wetter conditions, flooding and erratic rainfall leads to soil waterlogging and consequent rootrotting. Farmers spend a significant amount of time getting products to market, or obtaining replacement inputs. High water velocity and hailstorms reduce seedling survival, or leads to complete crop loss (57% of cases). When the rainfall arrive late, staple crops have fewer growing days so at the end of the season, have lower yields, do not ripen, or produce less seed. In hotter conditions livestock that provide meat, milk, fertilizer, transportation, and power experience cardiac arrest, heat stress, and “drooling disease.” In both in hotter and wetter conditions, market price volatility increases, and farmers generally have to pay higher prices for inputs, thereby reducing profit margins.

### Food security and health

**Fig. 3:**
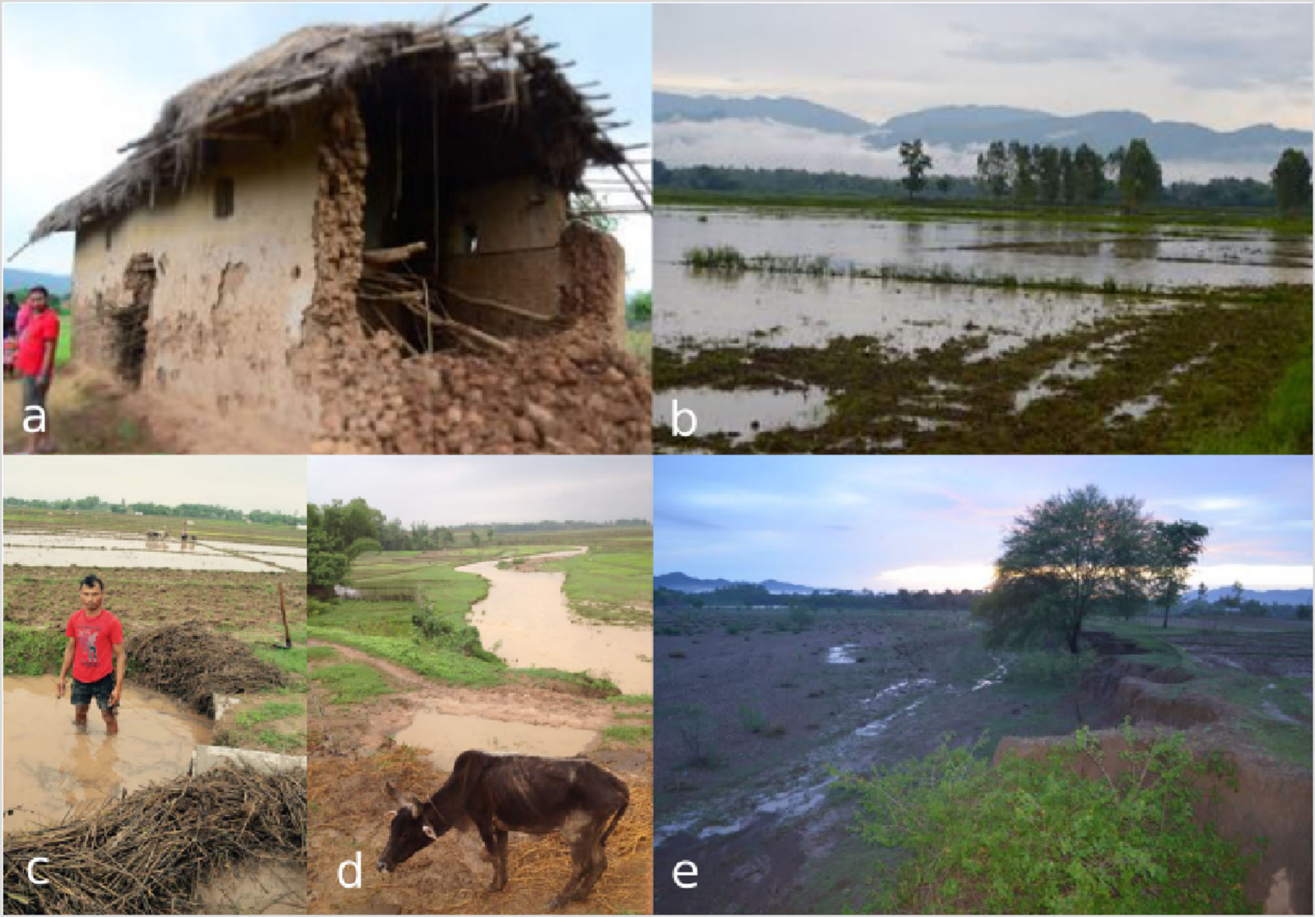
Images illustrating infrastructural damage and crop sedimentation from heavy rainfall and flooding in the Terai Plains of Nepal (July/August, 2012). **a** Damage to a communal grain store that collapsed after heavy rainfall in Kunjiwar, Duruwa VDC, Dang Valley. **b** Rice fields covered in sediment adjacent to breached riverbanks of the Tinau River in Makrahar VDC, Rupandehi district. **c** Obstruction of irrigation canals from debris after flooding in Manikapur, Bijauri VDC, Dang. **d** During heavy rainfall, flooding erodes riverbanks and increases river channel depth. Here, river water covers fields where rice seedlings are cultivated, leaving the land unproductive, livestock drowned, and crops lost in Lamaai, Dang. The change in the profile of the river leads to downstream flooding. **e** Productive land washed away and hundreds of hectares abandoned when a tributary of the Bagaai River diverted its course in the Dang District

In hotter, wetter conditions, 20% fewer farmers are able to get sufficient yield, while 27.5% fewer farmers are able to get sufficient food quality. Although levels of food availability are generally high (11.24 ± 0.37 months year^−1^, and 87% produce sufficient rice to feed their households year-round), scarce months typically fall at the end of the rainy season when stores are depleted, roads become inaccessible, and households must wait for harvests. As a result, food availability declines by 30%. Additionally, utilization is also affected. Following weather shocks in the preceding year, 43% study respondents reported they did not obtain sufficient nutrients from vegetables, fruit and meat. The spread of waterborne pathogens during flooding (e.g., cholera, diarrhea) more likely affects farmers who live close to open defecation areas or health facilities. Work productivity losses occur with heat stress, sunburn, disturbed sleep patterns, or heavy rain. Mortality and property damage are additional risks (Fig. 3).

### Adaptations to land management in response to biodiversity and climate change

To respond to biodiversity and climate change, farmers autonomously adapt land management at individual, household, and community levels. For example, Community Forest User Groups (CFUGs) and Buffer Zone Committees encourage sustainable utilization and equitable distribution of forest resources. CFUGs reforest to restore indigenous plant species, improve soil fertility, mitigate erosion, prevent flash flooding, and recharge water sources. CFUGs provide information about the access, use, and harvestability of forest products. They also monitor forest resources and the surrounding landscapes. To control invasive species, farmers primarily mechanically remove weeds, control fires, and mulch. Alternatively, they plant stale seedbeds, with narrow planting spaces between seedlings and flood-transplanted rice. Hand-held hoe and pulling were traditionally applied, but this is declining as it is considered labor intensive.

To improve equal access to water regulation and supply, local communities form Water User Associations (WUAs) which are responsible for building gabions, temporary check dams, bridges or concrete dikes, or installing boring pumps. These associations also form rules for water allocation, as well as regulate water velocity and silt load, divert river water, and maintain or improve irrigation infrastructure.

In hotter, wetter conditions, 10% more farmers apply chemical pesticides. Most (85%) rice farmers use readily available organophosphates, including *nuvan* (dichlorvos), *metacid*e (methyl parathion), *rogor* (dimethoate), and *unomide* (teriflunomide). Only 8% use organic pesticides, such as decayed leaves and seeds of neem (*Azadirachta indica*), Persian lilac (*Melia azedarach*), malabar nut (*Justicia adhatoda*), combined with ash and cow or buffalo urine. Few (15%) are able to actively manage natural enemies of pollinators, predators, decomposers, or parasitic wasps (e.g., *Halticoptera* sp., *Chrysocharis* sp.). They do this by planting wildflower strips, contour hedgerows, semi-natural, or set-aside habitats.

Farmers are aware that chemical pesticides can have adverse impacts on community composition of beneficial organisms (e.g., earthworms, amphibians), but generally (90%) lack knowledge about appropriate types, dosages, and timing of application (FAO, WFP, IFAD 2012). While many are aware that insecticides contaminate the soil and water, are costly (c. 719 NRS/year), and can be toxic if ingested (e.g., monocrotophos which is banned in the EU and US), few refer to the label for toxicity levels, use protective equipment for handling, actively manage residuals, or use planting methods to reduce secondary pests.

To manage plant hosts for fungal pathogens, 52% of farmers rotate crops. However, rotation is constrained by land scarcity and the need to replace expensive structures. Others apply fungicide, clean the host plant, treat the soil, and switch crops. To encourage genetic diversity, 69% of farmers grow a combination of local and hybrid rice varieties. Local varieties of seeds are shared through informal networks or stored in community seed banks. Farmer’s Field Schools (FFS) train land managers on location-specific in situ seed conservation. Cooperatives help to improve markets local crop varieties by building and maintaining roads, establishing collection centers, transporting produce, and providing price information.

To mitigate human–wildlife conflicts, farmers pool labor to guard their fields and property, while the government and NGOs install electric fences. Yet, park authorities often neglect livestock depredation, do not maintain fencing, and many areas remain unelectrified. To spread risk if one crop fails, farmers cultivate crops in different seasons, or diversify or replant crops planted in the same season. To improve water efficiency and retain soil moisture, farmers mulch, or spray water over saplings. To conserve water and decrease runoff and erosion, others construct trenches, raised or sunken beds, or stone bunds along contour lines. Riparian buffer strips shield against overland or shallow subsurface water flow from agricultural fields, provide habitat for declining fauna, and areas to grow plants for thatching, weaving, and brooms. To reduce heat stress, an isolated few farmers confine livestock in enclosures.

When experiencing food shortages, farmers eat fewer and smaller meals, comprising more affordable, low-quality foods, and/or produce food only for household consumption. New forms of livestock husbandry generates income to buy food, as does cultivating high-value crops (e.g., garlic, sunflower, eucalyptus, silk, calamine oil, and watercress). Increasingly, individuals are shifting to daily wage-earning occupations, or liquidate productive assets (e.g., land, livestock). However, these strategies have the potential to reduce future food production (Burke and Lobell 2010). Daily food sharing is common practice (93%), as are labor swaps (85%) in the form of direct reciprocal agreements, or partially paid in cash, food, or alcohol. To distribute losses, others cultivate on multiple pieces land, or intensify labor. It is important to note that only 40% rely on developed insurance markets, or can access financial loans (e.g., from cooperatives, microcredit schemes, or banks), while 60% rely on social networks. Finally, migration is an increasingly important strategy to directly return resources or income to fill production gaps in a timely manner: 67% of households indicated a member had left for work elsewhere in the preceding 6 months. Yet, it has also resulted in the shrinking of nuclear families—leaving women and elderly in rural homes with greater responsibilities.

## Discussion

The case of farmers in the biologically diverse Terai provides evidence of human adaptation to rapid biological, climatic and ecological change. It illuminates the ways in which societies continually innovate, experiment and adapt to meet their needs, maintain cultural identity, and shape the natural world. While the extent to which human adaptations have resulted in actual change, or reversed detrimental impacts of biodiversity change, goes beyond the scope of this paper, the study indicates that communities hold substantial knowledge of unprecedented changes in biodiversity and climate. Farmers can be seen to incrementally “adapt from below,” as species change in range, abundance, and phenology; new pathogens and weeds emerge; and subsequent changes occur in ecosystem functions and services. Ultimately, such strategies are responses to a multiplicity of sociopolitical–economic stresses (Mukul 2011; Thorn, Thornton and Helfgott 2015).

### Observed changes in species distribution and habitat across a climatic gradient

To our knowledge, this study provides the first evidence in the Terai of observed changes in species diversity with potential changes in climate. That is, significant differences were found across all climatic regions in plant diversity, and with a mean total precipitation reduction of 48 mm and mean annual temperature reduction of 2.98 °C. Nevertheless, climate risks arise from complex interactions between environmental, social and economic systems, so causality only due to climatic factors cannot be attributed to differences across sites.

### Comparison of farmers’ perceptions of changes and scientific observations

Some farmer perceptions are in close agreement with meteorological observations in Nepal—showing a maximum temperature rise of 0.04–0.06 °C year^−1^, decline in premonsoon precipitation, increase in postmonsoon/winter precipitation, and extremes in monsoon variability (IPCC 2014). However, other observations lack accuracy. For example, some farmers attribute longer-term change to what is seasonal variability (e.g., seasonal fluctuations in surface water). Many perceptions of changes in species distributions and habitat are equally supported by previous research (e.g., Akhalkatsi et al. 2017; Peniston 2013; Government of Nepal 2014b; Makul 2011).

### Limits to adaptations of autonomous land management

Adaptations are insufficient to maintain income, crop yields and safe living circumstances. Overall, farmers seem to be more likely to change irrigation technique or crop rotation schedule (incremental change), compared to changing crop variety (systemic change) or switch entirely to a nonagricultural livelihood or location (transformative change) (Meadu et al. 2015). The adoption of new strategies are limited by access to adequate credit, inputs and extension services. Additional capital and external assistance is typically required for large infrastructural investments and maintenance. High levels of malnutrition (43%) correspond with estimates that 36% of Nepali children under the age of 5 years suffer from chronic malnutrition, or stunting, among the highest rates in the world (World Bank 2015). Further, not all risk is equally distributed. Farmers that occupy land close to rivers, and cultivate low-lying crops are particularly susceptible to flood risk, while people who occupy slopes with unstable ground are exposed to landslides. Most of these farmers (87%) have insecure land tenure, which stems from a long history of exploitative tenancy relationships against certain castes, women, landless farmers, and ethnic minorities. Such insecure tenancy disincentives long-term management, while tenant agriculture is typically insufficient to support family cash or nutritional requirements (Appendix S7).

### Institutional arrangements and policies supporting extra-local adaptation

Given these limits, various new institutional arrangements are designed to aid farmers to adapt to biodiversity and climate change. Community-led institutions, such as CFUGs, WUA, and FFS, bring together groups to make rapid decisions to respond to change, manage or pool communal resources, build leadership, facilitate interaction, and demonstrate practices (Guneratne 2002). External agencies use these entities to channel resources, monitor ecological change, consult, or provide training and follow-on support. Ideally, these institutions account for local heterogeneity in groups and cultural values. However, distributional access challenges remain. At higher levels of governance, Nepal has recently adopted policies and strategies in line with international agreements to curve biodiversity loss [e.g., The Agriculture Development Strategy 2015–2035 (Government of Nepal 2015) and the National Agrobiodiversity Strategy and Action Plan 2014–2020 (Government of Nepal 2014a)]. Nepal was also one of the first countries to develop Local Adaptation Plans of Action for each of the 70 VDCs in 2012 and developed working groupism, such as “Forests and Biodiversity” and “Climate-induced disasters” (Government of Nepal 2010). Similarly, Nepal’s Poverty Reduction Strategy (2002–2007) recognizes the interdependence of ecosystem services, rural livelihoods, and agricultural systems. Various initiatives train farmers to conserve underutilized species and indigenous seeds, and increase market demand and nutritional awareness (e.g., National Agriculture Genetic Resources Centre). However, policy implementation has been weak, often not backed by legislation. National and local government measures have generally been reactive and insufficient to reduce farmers’ risk. Monitoring fine-scale dynamics of landscape change is rare, while frequent changes in government limit institutional memory (Sugden 2013). In sum, farmers usually adapt in unnoticed, uncoordinated, and unaided ways that are rarely reflected in formal mechanisms (Devkota et al. 2014). As such, existing autonomous adaptation priorities may hold the greatest potential, regardless of the political or institutional context.

### Implications for broader areas of integrated adaptation planning

To catalyze opportunities presented by human adaptation to biodiversity and climate change, the following five areas of consideration were identified during study interviews. These offer potentially insightful avenues for future policy development.

1. Prioritizing the needs of indigenous communities living adjacent to protected areas: Policies need to prioritize equitable access to natural capital, support rehabilitation and restoration in working landscapes that provide buffers against extreme events and essential provisioning services, and account for communities’ cultural heritage.
2. Low-cost labor-saving technologies and access to information and skills training: Increasing access to and use of such technology offers opportunity to enhance soil quality (e.g., threshers, mechanized plowing), sustain freshwater supply (e.g., microirrigation, solar or biodiesel water pumps), reduce deforestation (e.g., fuel-efficient cook stoves), maintain genetic diversity (e.g., seed storage facilities with electrical connections), and introduce high-yielding varieties adapted to new conditions. Additionally, by saving labor time, technologies can counter the rising workload of women, costs, and yield deficits, as well as reduce respiratory diseases and support youth remaining in or returning to rural areas. To maximize these opportunities, information and skills training is needed to adjust the timing of planting and varietal selection, for the safe and effective use of inputs, multiple-use tree planting, postharvest processing techniques, trap-/inter-/multi-cropping, parasite management, and eligibility requirements to access subsidies (FAO, WFP, IFAD 2012).
3. Information Communication Technology (ICT): Recent developments in ICT (e.g., mobile voice mail, text messaging, online training platforms, radio broadcasts) offer a rapid and affordable means to provide market information and early warnings for farmers, as well as aid biodiversity programming in schools, environmental monitoring, and loan or saving systems.
4. Co-existence of diverse public, private, and mixed extension service providers: Multiple service provision can improve access to information on the broad-based management of ecosystems services, and ways of expanding market share. For example, even though numerous organizations work in the Terai, only 20% of farmers have access to technical extension support and 11% to government subsidies. On the other hand, agro-vets are the main source of information for farmers (63%), and looking ahead, the role of the private sector in agricultural supply chains will become more pervasive (Ferroni and Zhou 2012). Expanding requirements to attain agro-vet licenses could leverage commercial players to provide extension support—if technical recommendations and product quality are appropriately monitored (Yadav et al. 2012).
5. Clarifying ambiguous tenure agreements and stronger regulation of tenancy relationships: This could help local communities have more autonomy in over land, production, and biodiversity management (Conway et al. 2015). Any intervention should avoid increasing dependence on external support or changing markets, and have an exit strategy, thereby leaving a local institutional mechanism that enables communities to serve their own needs (Bastakoti et al. 2016).

## Conclusions

This paper contributes to a growing body of theoretical and applied literature conceptualizing how biodiversity, climate, and human adaptation are specifically interrelated. Using the case of indigenous communities in four agroecological landscapes in the Terai Plains of Nepal, it offers three key contributions.

First, results show that compared to the global context, farmers are acutely aware of high levels of biodiversity and climatic change, and how this impacts ecosystem services and livelihoods. For example, 93% of farmers report that invasive species are proliferating in more humid, hotter conditions, and in locations where land is monocropped, cleared, or inputs are indiscriminately used. Ninety-eight per cent report that forest habitats are declining, thereby affecting areas used for hunting, or collecting water, NTFPs, and forage. In hotter conditions, quantities of irrigation and drinking water decline (by 40% and 20%, respectively), while the incidence of pests and plant hosts for fungal pathogens increases by 66%. Another 65% observe declines in genetic diversity, given the increasing temperature, changing preferences for hybrid characteristics, and habitat fragmentation, along with changes in the abundance and distribution of wildlife. Twenty-five key changes in climate were reported by 97.5% of farmers to reduce cultivated and livestock goods and services, and food self-sufficiency and security, and increase exposure to waterborne pathogens, heat stress, or mortality. Many of these perceptions of changes in species distributions, habitat, and climate are supported by previous research.

Second, evidence is provided of autonomous adaptations to land management “from below.” Existing incentives to conserve, restore, or sustainably manage ecosystems offer lessons for other societies undergoing rapid change, and dovetail inexorably with various targets of the post-2015 development agenda 2030 Sustainable Development Goals, *inter alia*, 2, 3, 6, 9, and 12 (UN General Assembly 2015). Nevertheless, adaptations are insufficient to maintain income, crop yields, and safe living circumstances. Adaptation strategies in one context may not be appropriate in every context, and tradeoffs need to be managed (Bhatta and Aggrawal 2016b). It is ever more evident that financial and institutional supports at national and regional levels are needed.

Finally, to support local adaptation in Nepal and elsewhere, the study illustrates the need to strengthen agroecological practices, upscale Information Communication Technology for extension services, clarify ambiguous tenure agreements, and safeguard natural ecosystems to enhance conservation beyond protected areas. In the wake of the post Paris Agreement, the Sendai Framework, and the Aichi Biodiversity Targets, a better understanding of how biodiversity and climate change interact is important to help societies to adapt to converging stressors, and slow biodiversity loss.

Future longitudinal research could monitor the impact of adaptation on marginal changes in forest or on-farm diversity, compare what factors influence adaptation within particular populations or households, or follow individuals as they move between rural and urban areas, adopt new technologies, incorporate new information, or recover from shocks (Bhatta and Aggarwal 2016b). Systematic assessments could compare impacts years of high productivity to years of deficiencies, investigate land history, compare anecdotal perceptions of species distribution and abundance to actual count data, and assess whether recommended policy approaches are still valid. Further participatory action-oriented research has an important role in jointly determining and implementing adaptation options that are feasible, effective, and carefully reviewed to avoid maladaptive outcomes.

## Electronic supplementary material

Below is the link to the electronic supplementary material.

Supplementary material 1 (PDF 864 kb)
