# Supplementary material for: Adaptation “from below” to changes in species distribution, habitat and climate in agro-ecosystems in the Terai Plains of Nepal
Source: Ambio. 2019 Jun 10;48(12):1482–97. doi: 10.1007/s13280-019-01202-0 (PMC6882764; doi:10.1007/s13280-019-01202-0)
Supplement: Supplementary file 1 — Supplementary material 1 (PDF 864 kb) [file 13280_2019_1202_MOESM1_ESM.pdf]

Ambio

Electronic Supplementary Material
This supplementary material has not been peer reviewed

Title: **Adaptation "from below" to changes in species distribution, habitat and climate in agro-ecosystems in the Terai Plains of Nepal**

Jessica P. R. Thorn

Appendix S1. Summary of representation from key informant interviews (*n*=174)

(VDC: Village District Committees, INGOs: International non-governmental organizations)

Level	No.	%	Sectors	No	%	Institution type	No	%
Local (VDC)	31	27	Agriculture and food security		36	Community representative	29	26
National	20	21	Development	36	32	Government	22	20
Local (Ward)	18	18	Environment and climate	20	18	NGO	17	15
District	18	13	Risk reduction	6	5	Private sector	15	13
International	14	12	Finance and business	6	5	INGO	14	13
Regional	11	9	Education	2	2	Research	12	11
			Gender and health	2	2	Donor or media	3	3

Appendix S2. Comparison of mean temperature and precipitation regimes and changes in each site over 20 years, in study sites in Nepal (meteorological trend data from regional synoptic stations, 1991-2011)

The table shows changes in climatic conditions over 20 years across climatic regions sampled in this study. The analysis considered total precipitation, minimum, maximum and annual temperature based on monthly data from regional synoptic stations. Comparisons were made of mean minimum, maximum and annual temperature over 20 years; mean annual precipitation for the current day; minimum, maximum and annual temperature change over 20 years; the rate of rainfall change over 20 years; and change in rainfall using mean value and the most recent year with a full dataset (2009). The greatest increases in mean annual temperature (°C) over the entire period were identified in Dang, while the highest increases per annum were identified in Deukhuri. The greatest increases in total annual precipitation (mm) both over the entire period and per annum were identified in Deukhuri (shown in bold). *Data for minimum/maximum temperature was unavailable for Rupandehi and synoptic data was not available for Chitwan.

**Climatic region**	**Mean current day (2009)**	**Mean per annum over entire period**	**Changes over entire period**	**Changes per annum using mean values**	**Change per annum using most recent year (2009 v 1991)**	**Change per annum using most recent year (various)**	**R^2 value**
	Total annual rainfall	*Total annual rainfall*	*Total annual rainfall*	*Total annual rainfall*	*Total annual rainfall*	*Total annual rainfall (2010 v 1991)*	*Total annual rainfall (1991-2010)*
Rupandehi*	1503.9mm	1623mm	143mm	**13.82 mm/a**	**7.53mm/a**	**14.59mm/a**	0.02461
Dang	1376.9mm	1575mm	61mm	13.62mm/a	3.21mm/a	13.34mm/a	0.05086
Deukhuri	1554.2mm	1598mm	**171 mm**	11.39mm/a	9.01mm/a	11.3mm/a	0.06299
	Mean annual temp	*Mean annual temp*	*Mean annual temp*	*Mean annual temp*	*Mean annual temp*	*Mean annual temp (2009)*	*Mean annual temp (1991-2010)*
Rupandehi	25.18mm	24.82°C	0.54°C	0.0114°C/a	0.017°C/a	0.017°C/a	0.26844
Dang	22.92mm	22.59°C	**0.98°C**	0.0113°C/a	0.028 °C/a	0.028 °C/a	0.19451
Deukhuri	22.71mm	21.84°C	0.57°C	**0.0055°C/a**	**0.051°C/a**	0.051°C/a	0.07979
	Mean min temp	*Mean min temp*	*Mean min temp*	*Mean min temp*	*Mean min temp*	*Mean annual min temp (2011 v 1991)*	*Mean min temp (1991-2011)*
Rupandehi	NA	NA	NA	NA	NA	NA	NA
Dang	16.49mm	16.10°C	0.05°C	-0.018°C/a	0.00263°C/a	0.27°C/a	0.38998
Deukhuri	16.15mm	15.62 °C	0.33°C	-0.01053°C/a	0.01754°C/a	0.06644 °C/a	0.01929
	Mean max temp	*Mean max temp*	*Mean max temp*	*Mean max temp*	*Mean max temp*	*Mean annual max temp (2011 v 1991)*	*Mean max temp (1991-2011)*
Rupandehi	NA	NA	NA	NA	NA	NA	NA
Dang	29.34mm	29.09°C	1.02°C	0.04°C/a	0.05°C/a	-0.006°C/a	0.05025
Deukhuri	29.27mm	28.09°C	1.62°C	0.02°C/a	0.09°C/a	0.0825°C/a	0.44307

Appendix S3. Semi-structured socio-economic and land management questionnaire

Socio-economic information

1. Full name of enumerator
2. Date
3. Name of village
4. Ward number
5. Village District Committee
6. District
7. GPS coordinates (WPM 84 format)
8. Elevation (masl)
9. Full name of respondent (representative of household)
10. Contact number
11. Home address
12. Age
13. Gender
14. Caste
15. Time lived in the community (years)
16. Description and sketch of field site

Farming system

17. Total area under cultivation (ha)?
18. Annual irrigated rice yield in the last season?
19. Amount of time land has been cropped (years)?
20. Most important crop cultivated?
21. Second most important crop cultivated?
22. Number of livestock units owned?
23. Type of livestock?
24. Land ownership agreement (select)? Owned / leased
25. Yield of rice (tonnes/ha/previous season).
26. Description of the seasonal cropping cycle.
27. Has the cropping pattern changed in the last ten years (since 2002)?
28. Percentage of crop used for different purposes (%) (household use, sale, fodder, fuel, gifting, other).
29. Key challenges to production.
30. What are the negative environmental impacts of cultivation of the key crop?
31. What are the negative health impacts of cultivation of the key crop?
32. What could be done to reduce these negative impacts?

Household characteristics

33. Livelihood activity(ies) (select)? Farmer / livestock rearing / tractor operation / teacher/ social mobilizer / politician/ community leader/ foreign employment / business ownership / aquaculture / agricultural extension / bee-keeping / wagon-driving / own a medical centre / other

34. Size of community/village (no. households)?
35. How long have you lived in the community (years)?
36. How did you come to live here (select)? Migrated from the hilly region/ born here/ bought uncultivated land/ bought cultivated land/ claimed forested land/ marriage/ received government subsidy to move here/ forcibly relocated/ other
37. Have you had a member leave for work elsewhere in the preceding six months?
38. What is the main source of energy for your household (select)? Firewood collected from community forests/ grid-connected electricity / solar power/ human or animal biogas/ battery/ kerosene lamps/ liquefied petroleum gas / ox or cow dung / crop residue / other
39. What is the energy used for (select)? Cooking / lighting / heating/ other
40. What is your home build with (select)? Local materials/ procured synthetic products/ both
41. Building material used?
42. Do you use wood as a main building material (select)? Yes/ no
43. What is the main source from where you get building material?
44. Have you noticed a change over time in availability of building material (select)? Yes/ no
45. What is this change caused by?
46. How months out of the last year did your family have sufficient income (select)? 0-2 months/ 3-5 months/ 6-9 months/ 9-12 months
47. What is your main means of transportation?
48. In the last week, did any one in your household share food with a member outside of your household?
49. In the last week, did any one in your household swap labour?
50. In the last week, did you get a financial loan?
51. Where did you get a financial loan? (E.g., insurance, cooperatives, village elders, micro-credit schemes, banks)
52. What is your main means of communication?

Perceptions of biodiversity change and adaptation strategies

53. Describe biodiversity changes you have observed in the last ten years (if any)?
54. What are the major drivers of these changes?
55. What are the associated risks for livelihoods?
56. What are the associated risks for crop and livestock production?
57. What are the associated risks for market access?
58. What are the associated risks for food security?
59. What are the associated risks for health?
60. What are the associated risks for water regulation and supply?
61. What are the associated risks for pest and disease regulation?
62. What actions do you employ at the local level and extra-local level to reduce adverse impacts to biodiversity change?
63. How do you adjust land management practices in response to biodiversity change?
64. What are the limits to these strategies?

Perceptions of environmental change and adaptation strategies

65. Compare the climate conditions to the last ten years with respect to mean and variance in precipitation and temperature.
66. Describe other climate-driven changes you have observed in the last ten years (if any)?
67. What are the associated risks for crop and livestock production?
68. What are the associated risks for livelihoods and market access?
69. What are the associated risks for food security and health?
70. What are the associated risks for irrigation water regulation and supply?
71. What are the associated risks for pest and disease regulation?
72. What actions do you employ at the local level and extra-local level to reduce adverse impacts to climate variability and change?
73. How do you adjust land management practices in response to climate variability and change?
74. What are the limits to these strategies?
75. Do you remember a time when this town was impacted by a major environmental shock such as flood, or drought?
76. If yes, please describe what happened and how you responded.
77. What other factors influence vulnerability to climate-driven changes?
78. How is risk differentiated (e.g., across groups, spatially, temporally)?
79. What are the main limits to adaptation?

Food security and health

80. What are the associated food security risks from climate variability and change?
81. What are the associated risks for health from climate variability and change, including work productivity, and waterborne diseases?
82. Has your household experienced crop loss in the last six months?
83. How many months of you are able to access food in a given year?
84. Is your household able to product sufficient rice year-round (select)? Yes/ No
85. When do months of food scarcity typically occur?
86. Was your household able to obtain sufficient nutrients (e.g., from vegetables, fruit and meat) following weather shocks in the preceding year (select)? Yes/ No
87. Are gender related concerns an issue to your or a member of your household (select)? Yes/ No

Wetland habitat and water regulation and supply

88. Does your household rely on rain-fed agriculture (select)? Yes/ No
89. What is your household's main source of irrigation water (select)? Rainwater tanks/ hand-drawn tube-wells/ electric tube-wells/ ponds/ canals/ electric pump/ diesel boring/ hand boring/ direct flow from rivers/ other
90. What is your household's main source of drinking water (select)? Rainwater tanks/ hand-drawn tube-wells/ electric tube-wells/ ponds/ canals/ electric pump/ diesel boring/ hand boring/ direct flow from rivers/ other

91. Is access to irrigation water communal or privately owned (select)? Communal / private
92. Is access to drinking water communal or privately owned (select)? Communal / private
93. If your household uses a tube well, state the depth in feet.
94. Is water extracted from shallow aquifers, surface water or both?
95. Has your household observed changes in irrigation water quantity in the last ten years (select)? Improved / same / declined
96. If yes, what are the reasons for these changes?
97. Has your household observed changes in irrigation water quantity in the last ten years (select)? Improved / same / declined
98. If yes, what are declines attributable to?
99. When is water scarcity the highest?
100. What are non-climate factors hindering water regulation and supply?
101. What is the quality of the water (select)? Improved / same / declined

Pest and disease regulation

102. What is the local name of pests found on your farms that are indicators of biodiversity and climate change? (free list)
103. What is the local name of pests found on your farms that have the most severe impact in terms of yield, income and household consumption? (Free list)
104. Which crop(s) do pests affect?
105. What is proportion of the crop(s) damaged from the pest (visual assessments) (%)?
106. Has the incidence and severity of pests changed in the last ten years (select)? (Increased/ same/ declined)
107. If yes, what is the reason for this change?
108. Has the incidence of invasive exotic species on farms changed in the last ten years (select)? (Increased/ same / declined)
109. What is the reason for this change?
110. What are the local names of new and invasive species found in and around your farm? (free list)
111. What are the new diseases you have observed in the last ten years?
112. What are the new pathogens you have observed in the last ten years?
113. Do you monitor the change in pests over time?
114. If yes, how?

Adaptations to land management and policy implications

115. What are your methods for pest management?
116. What chemical pesticides do you use?
117. What organic pesticides do you use?
118. Has the use of chemical pesticides change on your farm in the last ten years?
119. Do you perceive negative impacts of inorganic pesticide application on human health, ecosystem services or beneficial organisms of agro-ecosystems?
120. Do you actively manage pesticide residuals?
121. Do you observe growing pesticide residence or the emergence of secondary pests?

122. Do you rotate crops to reduce fungal pathogen spread?
123. Do you refer to the pesticide label for toxicity?
124. How much do you spend on pesticides per year (NRS)?
125. What type of fertilizers do you use (include ingredients)?
126. What type of pesticides do you use (include ingredients)?
127. Do you use of personal protective equipment and clothing in applying pesticides and fertilizers?
128. What is the ratio of NPK fertilizers you applied to the field in the last season?
129. Where do you get most of your information regarding the selection and other information on pesticide and fertilizer use?
130. What is your primary source of information about pest and disease management?
131. Do you manage for natural enemies of pests (select)? Yes/ no
132. If yes, what methods do you employ?
133. Do you apply any management practices to support pollination (select)? Yes/ no
134. If yes, what methods do you employ?
135. What are your methods for managing pathogens (select)? Yes/ no
136. If yes, what methods do you employ?
137. Do you apply any management practices to reduce weeds (select)? Yes/ no
138. If yes, what methods do you employ?
139. Do you apply any management practices to managing diseases, pathogens and parasites (select)? Yes/ no
140. If yes, what methods do you employ?
141. What variety (ies) of seeds do you use?
142. How many varieties of rice do you grow on your farm?
143. What is the reason for growing each rice variety?
144. Did you belong to a Community Forestry User Group in the last year (select)? Yes/ no
145. If yes, what were the benefits
146. Were you a member of a Water User Association in the last year (select)? Yes/ no
147. If yes, what were the benefits?
148. Were you a member of a farmer’s cooperative in the last year (select)? Yes/ no
149. If yes, what were the benefits?
150. Have you participated in a Farmer Field School in the last year (select)? Yes/ no
151. If yes, what were the benefits?
152. What information or skills training do farmers require?

Appendix S4. Villages included in this study with background information

[1]Coordinates and elevation of all villages were geo-referenced using a Garmin GPS62S and are shown in degrees, minutes and decimal minutes (UTM WGS 1984). [2] Due to transliteration, multiple spellings exist for villages.

District	Hydroshed	Tot. annual rainfall (mm)	Mean annual temp (°C)	Village[2]	Village District Committee	Ward	Coordinates [1]	Alt (m)	# of hh
Chitwan, Madi Valley	Rewu	2666	23.75	Gardi	Gardi	1	N27°28.305' E84°17.244'	188	200
				Gagoda	Baghauda	2	N27°27.462' E84°78.098'	168	300
				Shitalpur	Shitalpur	-	N27°26.758' E84°16.962'	167	42
				Laksmibas	Kalyanpur	3	N27°26.700' E84°20.390'	188	-
				Amelia	Gardi	1	N27°28.134' E84°17.100'	173	71
				Simara	Baghauda	-	N27°28.222' E84°20.429'	238	100
				Kharkatta	Ayodhyapuri	5	N27°28.221' E84°20.432'	210	148
				Bagai	Ayodhyapuri	9	N27°25.305' E84°26.739'	235	-
				Gaurinagar	Kalyanpur	9	N27°25.127' E84°27.135'	227	150
				Ganeshkunj	Ayodhyapuri	-	N27°25.482' E84°24.946'	255	150
Rupandehi	Tinau	1623	24.82	Beora	Makrahar	3	N27°35.414' E83°31.180'	118	100
				Tatera	Kerwani	6	N27°37.585' E83°33.400'	137	250
				Pedrani	Makrahar	8	N27°36.958' E83 32.287'	108	163
				Petbaniya	Kerwani	3	N27°35.028' E83°32.829'	127	200
				Bhutahawa	Sikhtahan	1	N27°37.588 E83°30.600'	125	46
				Katahani	Makrahar	6	N27°38.294' E83°31.509'	122	100
				Gorkhatta	Anandaban	8	N27°37.833' E83 26.732'	117	250
				Manigram	Anandaban	2	N27°37.400' E83°27.465'	173	-
				Semari	Tikulgadh	8	N27°28.061' E83°47.123'	215	215
				Shankarnagar	Dingarnagar	6	N27°39.213' E83 27.342'	133	361
Dang, near Ghorahi	Rapti	1598	24.82	Keruniya	Sonpur	2	N27°50.226' E82°36'090'	353	158
				Madehe Nagar	Chalahi	4	N27°50.783’ E82°30.068’	261	119
				Ghumna	Chalahi	6	N27°50.514' E82°32.599'	230	138
				Manicapur	Godhawa	5	N27°47.039' E82°32.449'	246	-
				Khairah	Gabadiya	9	N27°48.860' E82°36.528'	261	122
				Satbariya	Satbariya	5	N27°48.838' E82°36.553'	236	300
				Bagrapur	Sisaniya	9	N27°49.672' E82°38.207'	250	214
				Bangau	Satbariya	1	N27°51.163' E82°29.223'	249	130
				Motipur	Gadhawa	6	N27°49.690' E82°31.385'	241	26
				Gadhawa	Gadhawa	2	N27°48.646' E82°33.087'	241	85
Deukhuri Valley, Dang	Bogai	1575	22.59	Kunjiwar	Duruwa	7	N28°03.086' E82°18.712'	580	-
				Beldana	Duruwa	-	N28°02.748' E82°19.745'	567	12
				Jinnywa Gaun	Manpur	1	N28°05.147' E82°20.426'	606	120
				Aankani	Duruwa	8	N28°01.437' E82°17.792'	607	45
				Bigauri	Bijauri	5	N28°06.600' E82°20.088'	651	300
				Fulbari	Bakhariya	6	N28°02.091' E82°15.047'	546	179
				Kurahauria	Duruwa	6	N28°03.579' E82°17.991	594	100
				Jamera	Manpur	8	N28°03.430' E82°21.347'	601	30
				Manikapur	Bijauri	3	N28°06.531' E82°22.206'	658	400
				Karmatuwa	Dhitpur	6	N28°01.841' E82°21.250'	565	80

Appendix S5. Meteorological data

In Nepal, meteorological data was sourced from the Nepal Department of Hydrology and Meteorology in Kathmandu for Rupandehi and the synoptic station from the Mid and Far Western Regional Climate office for Dang and Deukhuri. For Deukhuri, the data originated from the synoptic station in Tulsipur Dang (N 28°08' E082°18', 725masl) located 11.1km north of and 128m higher than Kunjiwar (N28°03.086' E082°18.712’, 597masl). For Dang, the data originated from the synoptic station of Ghorahi Dang (N 28°03' E082°30', 634masl) located 32km south of and 378m higher than Chalahi, Madahe Nagar (N27°50.783’ E082°30.068’, 256masl). For Rupandehi, the data originated from the Bhairahawa airport meteorological station (N27°30’19.3’’ E083°25’10.5’’,109masl) located 13.85km southwest of and 29m lower than Beora, Rupandehi district (N27°35.414' E083°31.180', 138masl). Meteorological data for Madi Valley, Chitwan was not available from regional synoptic stations. In Dang rainfall data came from five stations, namely Nayabasti, Ghorahi (Masina), Ghorahi (Dang), Tulsipur and Koilabas. In Rupandehi, rainfall data came from four stations, namely Butwal, Bhairahawa airport, Bhairahawa agricultural station and Lumbini Mandir.

Appendix S6. Overview of plant families identified

The most dominant plant families are *Euphorbiaceae* (5 families), *Fabaceae* (4), *Moraceae* (4), *Anacardiaceae, Lamiaceae*, and *Rutaceae* (3). The most common species are *Shorea robusta* (6.7%), *Dalbergia sisoo* (Indian rosewood) (6.4%), *Azadirachta indica* (Mugwort) (6.4%)*, Melia azedarach* (Persian lilac) (5.1%)*, Leucaena leucocephala* (Leucaena) (4.4%), *Ficus religiosa* (Banaya tree) (4.4%)*, Dendrocalamus strictus* (Bamboo) (4.4%)*, Ocimum tenuiflorum* (Holy basil) (4.4%)*, Magnifera indica* (Mango) (3.9%), and *Jatropha curcas* (Physic nut) (3.1%).

Appendix S7. Brief history of land insecurity in the Terai

Typically, a cultivator (“*bahariya”*) enters into a three- to five-year contract with a landlord (“*raiti”*), who provides labor in return for access to the land, 50% of the produce, and farming inputs. Absentee landlords have a limited role in encouraging innovation or developing production forces. “*Landless*” farmers do not have official title deeds, may have lost land, be reluctant to obtain title for fear of taxation, be political migrants, or be categorized as being of a “lower” caste. Despite their long history of semi-nomadic cultivation in the Terai, many Tharus are landless. Many Tharus had no formalized ownership during the government-induced resettlement and sedentarization program, and were evicted or forced to sell their land to repay debt. Women similarly typically do not have land title deeds, and 87% ranked gender inequality as a major concern.
